# EMT/MET plasticity in cancer and Go-or-Grow decisions in quiescence: the two sides of the same coin?

**DOI:** 10.1186/s12943-023-01793-z

**Published:** 2023-05-31

**Authors:** Azamat Akhmetkaliyev, Noura Alibrahim, Darya Shafiee, Eugene Tulchinsky

**Affiliations:** 1 Department of Biomedical Sciences, Nazarbayev University School of Medicine, Astana, 020000 Kazakhstan; 2 Al-Mana College for Medical Science, Ahsa, Saudi Arabia; 3 Department of Genetics and Genome Biology, University of Leicester, Leicester, UK

**Keywords:** Cancer, EMT, MET, Stem cells, Quiescence, Cancer stem cells

## Abstract

Epithelial mesenchymal transition (EMT) and mesenchymal epithelial transition (MET) are genetic determinants of cellular plasticity. These programs operate in physiological (embryonic development, wound healing) and pathological (organ fibrosis, cancer) conditions. In cancer, EMT and MET interfere with various signalling pathways at different levels. This results in gross alterations in the gene expression programs, which affect most, if not all hallmarks of cancer, such as response to proliferative and death-inducing signals, tumorigenicity, and cell stemness. EMT in cancer cells involves large scale reorganisation of the cytoskeleton, loss of epithelial integrity, and gain of mesenchymal traits, such as mesenchymal type of cell migration. In this regard, EMT/MET plasticity is highly relevant to the Go-or-Grow concept, which postulates the dichotomous relationship between cell motility and proliferation. The Go-or-Grow decisions are critically important in the processes in which EMT/MET plasticity takes the central stage, mobilisation of stem cells during wound healing, cancer relapse, and metastasis. Here we outline the maintenance of quiescence in stem cell and metastatic niches, focusing on the implication of EMT/MET regulatory networks in Go-or-Grow switches. In particular, we discuss the analogy between cells residing in hybrid quasi-mesenchymal states and G_Alert_, an intermediate phase allowing quiescent stem cells to enter the cell cycle rapidly.

## Introduction

Epithelial mesenchymal transition (EMT) is a process of conversion of polarised epithelial cells into mesenchymal cells. First discovered in embryonic development, EMT and a reverse process of mesenchymal epithelial transition (MET) were later implicated in human diseases, organ fibrosis, and cancer. EMT and MET are vital in embryogenesis for the early (gastrulation, neural crest delamination) and late (formation of various organs, such as the heart) stages. The classical physiological function of EMT in normal and pathological conditions is the generation of motile cells capable of degrading and crossing basement membranes [1]. A basement membrane is a specialised extracellular matrix built by proteins deposited by epithelial cells and cells of the underlying stroma. It is composed of polymerized laminin, glycoproteins, and collagen fibrils and separates epithelial layers from stromal tissues [2]. After passing through basement membranes, cells undergoing an EMT may penetrate surrounding tissues and migrate to new destinations.

Epithelial cells are linked to the basement membranes via focal adhesions and epithelium-specific adhesion structures, hemidesmosomes. The structural integrity and functionality of epithelial tissues depend on intercellular adhesion complexes, tight junctions, adherens junctions (AJ), and desmosomes, which mediate attachments between adjacent cells [3]. These differentiated, highly specialised structures are essential for maintaining the basolateral polarity of epithelial cells and tissue homeostasis [1].

**Fig. 1:**
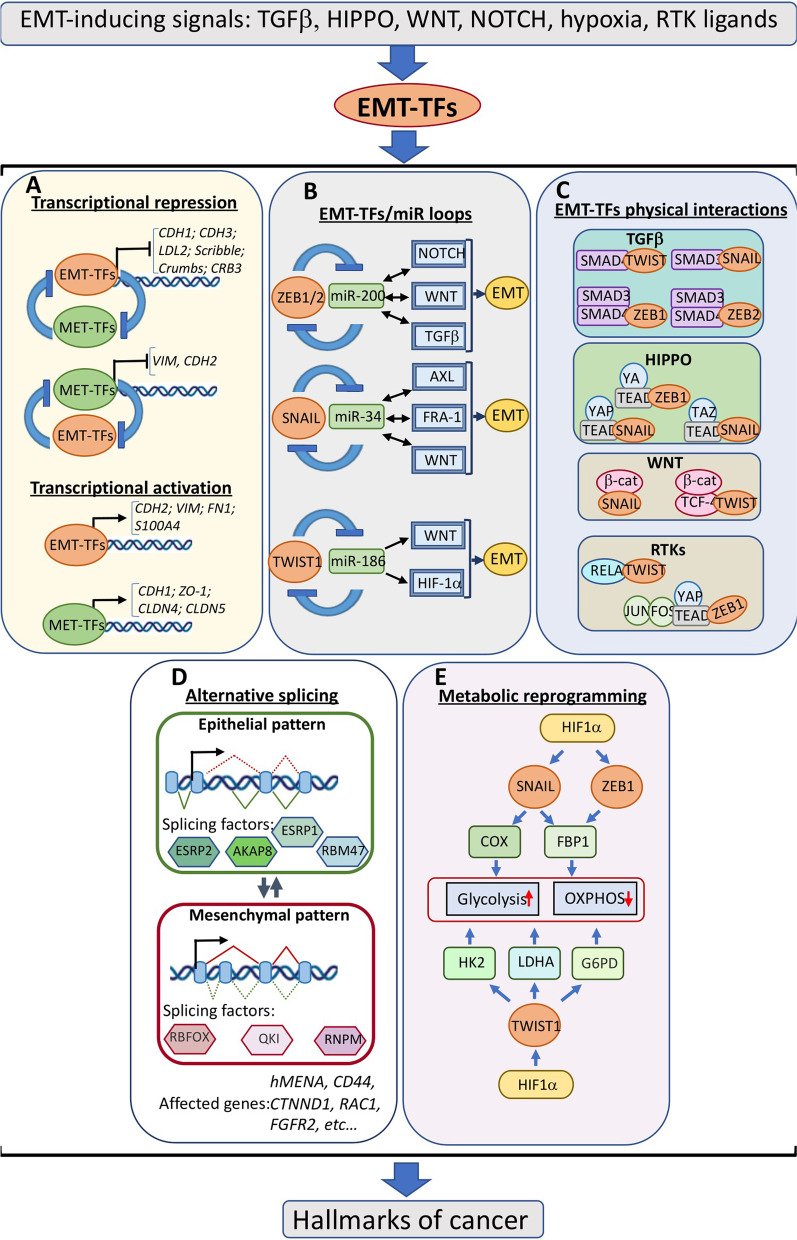
EMT pathways are embedded in signalling networks in cancer cells. Various signalling pathways exemplified on the top promote EMT by activating EMT-TFs. **A** Transcriptional regulation of epithelial-mesenchymal plasticity. In cells undergoing EMT, EMT-TFs repress transcription of epithelial genes encoding components of various epithelial structures, such as polarity complexes and adherens junctions, and activate the expression of mesenchymal genes. MET-inducing transcription factors (MET-TFs) repress transcription of mesenchymal markers, activate epithelial transcription programs, and act in double-negative feedback loops involving EMT-TFs. **B** EMT-inducing signals are modified by EMT-TFs/microRNA loops. EMT-TFs and microRNAs form interrelated double-negative feedback loops which affect expression levels of certain components of EMT-inducing signalling network. **C** EMT-TFs physically interact with components of signalling pathways forming complexes that in turn influence target genes. **D** EMT/MET mutually regulate alternative splicing. EMT-TFs regulate expression of epithelial (e.g., ESRP1/2) or mesenchymal (e.g., QKI) splicing factors, which in turn determine formation of epithelial- or mesenchymal-specific protein isoforms required for the accomplishment of EMT or MET programs. **E** Expression of metabolic genes of glycolysis and oxidative phosphorylation pathways are controlled by EMT-TFs

During EMT and MET, cells experience drastic changes in their physiology. In the course of EMT, the alterations involve the disappearance of intercellular adhesions, loss of epithelial polarity, dynamic restructuring of the cytoskeleton, degradation of basement membranes, etc. Several transcription factors collectively termed EMT-TFs govern the coordinated changes in cell physiology manifesting EMTs. Zn-finger transcription factors of SNAIL (SNAIL, SLUG) and ZEB (ZEB1, ZEB2) families and basic helix-loop-helix proteins TWIST1 and TWIST2 are best-studied EMT-TFs in the context of normal and pathological conditions [4–6]. Mechanistically, EMT-TFs repress transcription of a set of genes, whose function is critical for maintaining the epithelial state. Repression of the E-cadherin-encoding *CDH1* gene results in the dissolution of AJ; reduction in the levels of the polarity complexes components, Crumbs and Scribble, CRB3 and LDL2 (Lethal Giant Larvae 2) leads to the loss of basolateral polarity [7, 8] (Fig. 1A). EMT-TFs repress transcription by recruiting various co-repressor complexes with HDAC and HMTase activities, Mi2/NuRD/G9A [9, 10], SIN3a/HDAC1/HDAC2 [11], coREST/CtBP1/HDAG2/G9A [12], SWI/SNF [13], and EZH2/HDAC1[2] [14] among others (reviewed in [15]). Concurrently, EMT-TFs directly or indirectly activate transcription of mesenchymal genes, including a critical component of mesenchymal intermediate filaments vimentin, *CDH2* (codes for N-cadherin), matrix metalloproteinases specializing in the degradation of the basement membrane, and others. Direct activation requires binding co-activators with HAT activities, CBP, p300, and PCAF [15–18]. The binding of EMT-TFs to the effectors of some signal transduction pathways may result in their conversion from transcriptional repressors to activators (see Sect. 2). The EMT/MET balance is regulated by yet another group of transcription factors, which can be collectively referred to as MET-TFs. This relatively less explored set of factors includes diverse proteins belonging to various families, namely, zinc-finger-containing proteins Ovo-Like Transcriptional Repressor 2 OVOL2 [19] and Krüppel-like factor 4 (KLF4) [20], Grainyhead-like protein GRHL2 [21], and ETS family members E74-like ETS transcription factor 3 and 5 [22, 23] among others. MET-TFs promote epithelization in normal physiological conditions and cancer cells by direct transcriptional repression of mesenchymal markers and by establishing mutual bidirectional inhibitory circuits with EMT-TFs [24–27]. Moreover, some MET-TFs directly activate transcription of the genes encoding proteins specifying epithelial lineages, such as *CDH1*, *ZO-1*, *CLDN4*, and *CLDN5* (encode Claudin-4 and -5) [28, 29] (Fig. 1A).

### EMT and cellular signalling networks

EMT-TFs are induced in response to various signalling cues in embryonic or fibrotic tissues or tumour microenvironment, such as TGFβ, RTK ligands, HIPPO, WNT, NOTCH, or inflammatory cytokines [5, 30]. Importantly, EMT programs often modify EMT-inducing signal transduction pathways by activating positive or negative feedback regulatory loops. The underlying mechanisms vary in their nature. EMT-TFs form double negative self-enforcing loops with several classes of microRNA (Fig. 1B). For instance, ZEB proteins are repressors of microRNA clusters encoding miR-200 family members, which in turn are ZEB repressors [31, 32]. Similar connections exist between miR-34a and SNAIL family members and miR-186 and TWIST1. ZEB1/2-miR-200 module affects expression levels of critical components of TGFβ[33, 34], WNT [35–37], and NOTCH (up-regulation of MAML2/3; [38]) signalling pathways. When the balance in ZEB/miR-200 equilibrium is moved towards EMT-TFs, this may lead to the activation of TGFβ, WNT, or NOTCH pathways and thereby potentiate EMT via positive feedback loops. Likewise, SNAIL- or SLUG-induced EMT programs involve similar feedback regulation: repressing miR-34a subsequently leads to the increased expression of its EMT-potentiating targets, such as TGFβ1, LEF1, AXL, FRA-1, etc. [39–42]. TWIST1-targeted miR-186 is a repressor of the WNT pathway as well as HIF1α, among other targets known to contribute to the EMT programs in various cancer types [43, 44] (Fig. 1B). Long non-coding RNAs (lncRNAs) represent one more class of RNA molecules closely associated with regulating the EMT/MET balance. Various lncRNA species modulate EMT pathways via different mechanisms and at different levels. For example, lncRNA-ATB or lncRNA-OC1 may act as competing endogenous RNAs (ceRNAs) to promote EMT by sponging miR-200 family members or miR-34a respectively [45, 46]. Additionally, lncRNAs may serve as scaffolds for essential interacting components of EMT pathways or participate in the recruitment of chromatin-modifying complexes to the promoters of genes implicated in EMT or MET programs. The interactions of lncRNA with various signalling pathways implicated in epithelial plasticity are covered in recent comprehensive reviews [47, 48].

EMT-TFs also interfere with cellular signalling networks via direct physical interactions with the effectors of TGFβ, RTK, HIPPO, and WNT pathways (Fig. 1C). The resultant complexes recruit chromatin modifiers and ensure cooperative transcriptional control of target genes (reviewed in [49]. In particular, TGFβ-induced EMT programs involve physical and functional interactions between transcription factors SMAD2/3 and ZEB proteins or SNAIL [50, 51] or between TWIST1 and SMAD4 [52]. Binding of HIPPO effectors YAP and TAZ to SNAIL family members [53] or formation of multimeric complexes composed of sequence-specific transcription factors of TEAD, AP-1, NF κB families, and EMT-TFs [54–56] are essential for cooperation between EMT, HIPPO and RTK signalling. Binding the HIPPO effector YAP and AP-1 complexes results in the release of CtBP, and recruitment of HAT CBP and shift of ZEB1 from transcriptional repression to activation [54]. Complexes containing SNAIL and β-catenin were reported in colorectal cancer cells, and this interaction was essential for activating WNT-dependent genes [57]. TWIST1 was co-immunoprecipitated with TCF4/β-catenin complex in dermal papilla cells [58] (Fig. 1C).

The above-cited reports exemplify interactions between EMT and several signal transduction pathways, but they do not cover the impact of EMT on gene regulation in its full complexity. Indeed, EMT-microRNA circuits deeply interfere with fundamental cell signalling mechanisms at several levels. EMT pathways activate mesenchymal splicing programmes [59, 60] and are implicated in metabolic reprogramming [61, 62] (Fig. 1D, E). EMT is responsible for the rewiring of RTK signalling [63]; it alters the cellular epigenetic landscape [64]. Through the analysis of current literature and public databases, a recent study identified 932 genes in total that are associated with EMT/MET across various cancer types [65]. Therefore, it is not surprising that in addition to the classical role of EMT/MET programmes in determining phenotypic cell plasticity, they affect most, if not all, cancer features. These features classified as “hallmarks of cancer” by R Weinberg and D Hanahan include cell cycle regulation, replicative immortality, apoptotic resistance, immune evasion, evading growth suppressors, self-renewal ability, neovascularization and altered energetics (Fig. 1) [66].

The deregulated cell cycle and acquired cell stemness are among cancer characteristics closely associated with EMT/MET-controlled cell plasticity and will be discussed in the following sections.

### Go-or-Grow: EMT and cell cycle control in normal tissues

**Fig. 2:**
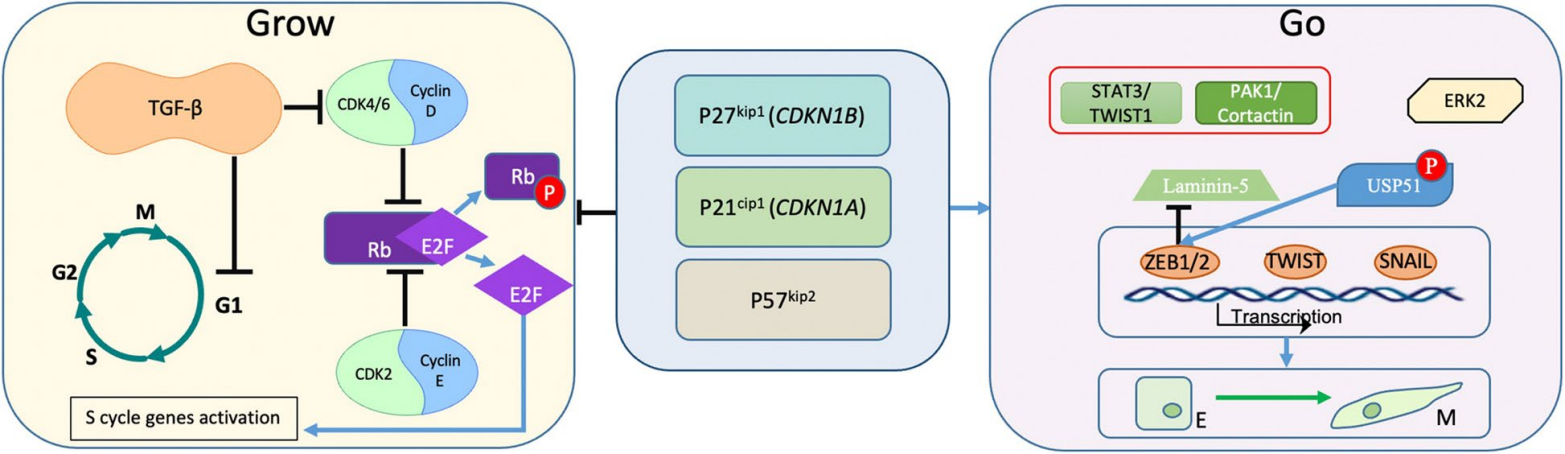
An overview of “Go-or-Grow” concept. Main orchestrators of CDK activity namely p27^KIP1^, p21^CIP1^, and p57^KIP2^ inhibit proliferation (”Grow”) and activate migration (”Go”) via direct/indirect interactions with the components of cell cycle machinery and EMT-TFs network (see text for details)

From a general perspective, molecular mechanisms controlling cell proliferation and migration must be connected. Indeed, migrating and mitotic cells share common signalling modules and structural elements (e.g., microfilaments and microtubules). The critical pathway which integrates growth-promoting and growth-suppressing signals into a decision for proliferation or cell cycle exit is Rb-E2F. Cyclin D-CDK4/6 and cyclin E-CDK2 complexes consecutively phosphorylate RB to drive G_1_ progression and entry into the S phase. Cyclin-dependent kinase inhibitors (CKIs), p27^KIP1^, p21^CIP1^, and p57^KIP2^, are key regulators of CDK activity [67] (Fig. 2). In addition to their canonical function in cell cycle regulation, CKIs are also implicated in cytoskeletal dynamics, cell migration, and invasion [68]. For instance, the central inhibitor of G_1_-S transition, p27^KIP1^, facilitates cell motility and invasion by increasing the turnover of invadopodia through PAK1/cortactin signalling [69, 70], affecting actomyosin dynamics [71], and by inducing EMT via STAT3-TWIST1 pathway [72] (Fig. 2). As cell migration and proliferation share common elements of molecular pathways, there might be competition in determining which process prevails in a given cell. Indeed, the mathematical modeling predicts that the increase in cell proliferation occurs at the expense of cell migration and vice versa [73]. This popular concept of the dichotomy between proliferation and migration is discussed in the literature as the “Go-or-Grow” or “Divide or conquer” concept.

The Go-or-Grow hypothesis was studied in normal physiological conditions. *C. elegans* anchor cells (AC) represent a model allowing to study molecular mechanisms of invasion in embryonic development. AC, specialized uterine cells, play a role in the development of the nematode reproductive system; they breach the basement membrane separating the uterine and vulval tissues to form a uterine-vulval connection. AC invasion depends on the* fos-1a* gene, whose loss prevents the disintegration of the basement membrane [74]. Of note, mammalian orthologues of fos-1a, AP-1 family members cooperate with EMT-TFs at different levels and are potent EMT inducers in various cancers [75–77]. Mechanistically, a nematode p21^CIP1^ homolog was required for the G_1_/S cell cycle arrest and, at the same time, essential for AC invasion [78].

An association between embryonic EMT programmes and cell cycle control was studied in the course of the formation of neural crest cells (NCC). This population of multipotent cells is generated via EMT during the closure of the neural tube in the embryonic development of vertebrates. The population of NCC gives rise to various cell lineages, including peripheral neurons, Schwann cells, melanocytes, and craniofacial structures, among others. Delamination of NCC from neural folds and their migration to new embryonal territories is regulated by distinct EMT-TFs in distinct species of vertebrates [79, 80]. It has been reported that different NCC utilize different mechanisms of EMT upon delamination, and their migration may or may not be associated with the exit from the cell cycle. While cranial NCC exhibit various distributions over the cell cycle phases, delaminating cells at the sites of neural tube closure within the trunk are accumulated in the G_0_/G_1_ phases (reviewed in [80, 81].

A mechanistic link between reversible cell cycle arrest (cell quiescence) and EMT was established in vitro in studies analyzing gene expression signatures of serum-deprived quiescent primary fibroblasts. In addition to a number of genes associated with the cell cycle regulation, serum deprivation activated several genes implicated in EMT, namely *TGFb1*, *IL6*, *IL11*, N-cadherin encoding gene *CDH2*, EMT-TF-encoding gene *ZEB2*, and others [82].

### Go-or-Grow: EMT and cell cycle control in cancer

The applicability of the Go-or-Grow concept to cancer is of particular interest. Indeed, on the one hand, a central feature of cancer cells is their ability to overcome growth-inhibiting stimuli and sustain uncontrolled proliferation [66]. On the other hand, most conventional or targeted anti-cancer agents are effective against cells passing through the S and G2/M phases of the cell cycle, and cancer cells residing in G_1_/G_0_ are therapy-resistant. Therefore, the proliferative plasticity or the ability of cancer cells to transit between proliferative and quiescent states poses an obstacle to therapy, and understanding underpinning mechanisms is critically important [83, 84].

EMT substantially impacts drug resistance largely because it is commonly associated with slow proliferation or cell cycle arrest. Indeed, one of the most potent EMT inducers, TGFβ, is a classical tumour suppressor and canonical inhibitor of the cell cycle in the G_1_ phase. TGFβ-mediated cell cycle arrest involves SMAD-mediated activation of *CDKN2B* and *CDKN1A* genes encoding CDK inhibitors p15^INK1^ and p21^CIP1^ [85]. In squamous cell carcinoma, TGF-β/SMAD signalling induced quiescence via activation of p21^CIP1^ in the proportion of cells. In contrast to the bulk of the tumour, these cells exhibited resistance to DNA damage and increased tumorigenic potential [84]. This study did not address the implication of EMT-TFs, which act in concert with TGFβ/SMAD. However, in earlier reports, the ability of EMT-TFs to attenuate G_1_/S cell cycle progression has been shown in vitro. In particular, SNAIL- and ZEB2-induced EMT in cultured carcinoma cells went along with the delays in G_1_- to S-phase transition caused by the transcriptional repression of *CCND2* and *CCND1,* respectively [86, 87]. In addition, CDK inhibitors are transcriptional targets of EMT-TFs; SNAIL activated p21^CIP1^-encoding gene *CDKN1A* [86]; and SLUG induced both *CDKN1A* and *CDKN1B* (codes for p27^KIP1^) in cervical carcinoma cells [88]. Concomitant with the attenuated cell cycle progression, EMT-TFs stimulate individual cell motility and invasion. To our knowledge, the impact of EMT-TFs-induced CDK inhibitors on the motility of cells during EMT has not been addressed. Activation of the G_1_/S checkpoint represents a common EMT feature; ERK2 induces EMT, which is associated with enhanced expression of *CDKN1A* and *CDKN1B* [89]. Emerging evidence indicates a negative feedback mechanism links EMT-TF ZEB1 with the G_1_/S checkpoint regulation. CDK4/6 kinases phosphorylate and stimulate deubiquitinase USP51, which stabilises ZEB1 [90] (Fig. 2). This regulatory circuit may fine-tune the balance between “Go” and “Grow” types of cell behaviour, and generate proliferating cells which an intermediate epithelial-mesenchymal phenotype (see following sections).

Experimental data supporting the “Go-or-Grow” hypothesis in cancer cells in vitro align with the analyses of patients’ samples. In several cancer types, high cell proliferation indices are typical for the central areas of solid tumours, whereas cells at the tumour periphery are often non-proliferative and highly invasive. According to some reports, enhanced expression of D- and E-type cyclins in breast cancer correlated with a less malignant phenotype and a more favourable prognosis [91, 92]. EMT at the invasive front of colorectal cancer is associated with the enhanced expression of the *CDKN2A* gene product p16INK4A, which correlates with low survival rates [93]. EMT programs at the tumour-stroma boundary are induced by ZEB1 and lead to the transient loss of the basement membranes due to proteolytic cleavage of their components and down-regulated synthesis of laminin-5, a basement membrane component secreted by tumour cells [94]. These data can be interpreted as proof of the dichotomy between the growth of primary tumours versus invasion, with the latter being responsible for poor prognosis. The “Go-or-Grow” phenotypic switch in cancer was modeled in vivo by knocking out the p21^CIP1^-coding gene *CDKN1A* in the polyoma virus middle T mammary tumour model [95]. Depletion of this potent CDK inhibitor stimulated tumour growth and resulted in the suppressed cell invasion in vitro and reduced metastatic spread. These results reflect the function of the nematode p21^CIP1^ homologous protein in the invasion of AC cells in *C. elegans*. They suggest that metastatic cancer cells can hijack and utilize the evolutionarily conserved mechanisms regulating the “Go-or-Grow” switches. On the other hand, there is no correlation between cell motility and proliferation in established cancer cell lines in vitro [96]. Therefore, mechanisms coupling cell cycle regulation and cell motility can be circumvented in cancer. Alternatively, the mechanisms maintaining “Go-or-Grow” switches do exist; but in a single cell, the “Go” and “Grow” functions are separated in time. On the level of cell populations, rapidly dividing cells can be highly invasive. These cell populations may exist in brain tumours, malignant mesothelioma, or cutaneous melanoma. Although the death of patients with these cancer types is caused mainly by metastatic disease, the unfavorable prognosis is closely associated with high mitotic activity [96]. This contrasts with the above-cited reports on the presence of slowly proliferating cells on the invasive front predicting poor survival of ER + breast or colorectal cancer patients [91–93]. If proliferative capacity and invasive potential within a given cell are not separated in time, this may have important implications for tumour biology. Cells that underwent TGFβ-induced EMT but were resistant to TGFβ-imposed growth inhibition displayed genetic instability and defects in cytokinesis. These abnormalities were associated with SNAIL-dependent suppression of Lamin B1 and other nuclear envelope proteins implicated in mitotic control. Thus, breaching the Go-or-Grow principle in cancer cells may trigger heritable genetic defects, subsequently contributing to tumor evolution [97].

### Quiescence, adult stem cells, and EMT

Quiescence is a characteristic of subpopulations of adult stem cells (ASC), a specialised type of tissue-resident cells responsible for tissue homeostasis and repair. In the epidermis, intestinal epithelium, and regenerating tissues, quiescent ASC co-exist with the stem cells repeatedly undergoing asymmetric divisions. The latter subpopulation maintains tissue integrity by differentiating into parenchymal cells and producing new stem cells (self-renewal) [98, 99]. Unlike irreversibly arrested senescent and terminally differentiated cells, quiescent ASC can be reversibly activated by environmental triggers [100, 101].

Stem cells are located in specialised niches formed by stem cell progeny, and supportive stromal cells. The niche determines a balance between quiescent and proliferating stem cells. Disruption of this equilibrium in a healthy tissue may result in deregulated cell proliferation, compromised tissue repair, impaired homeostasis, and regeneration processes [100]. For example, failure to maintain quiescence in hematopoietic stem cells (HSC) leads to the extended expansion of the stem cell population and its exhaustion leading to the depletion of adaptive immunity and myeloproliferative disorders [102]. The importance of quiescence was also demonstrated in the studies on NCC, the decrease in the hippocampal neural stem cell population resulted in cognitive defects due to decreased amounts of new neurons [103].

**Fig. 3:**
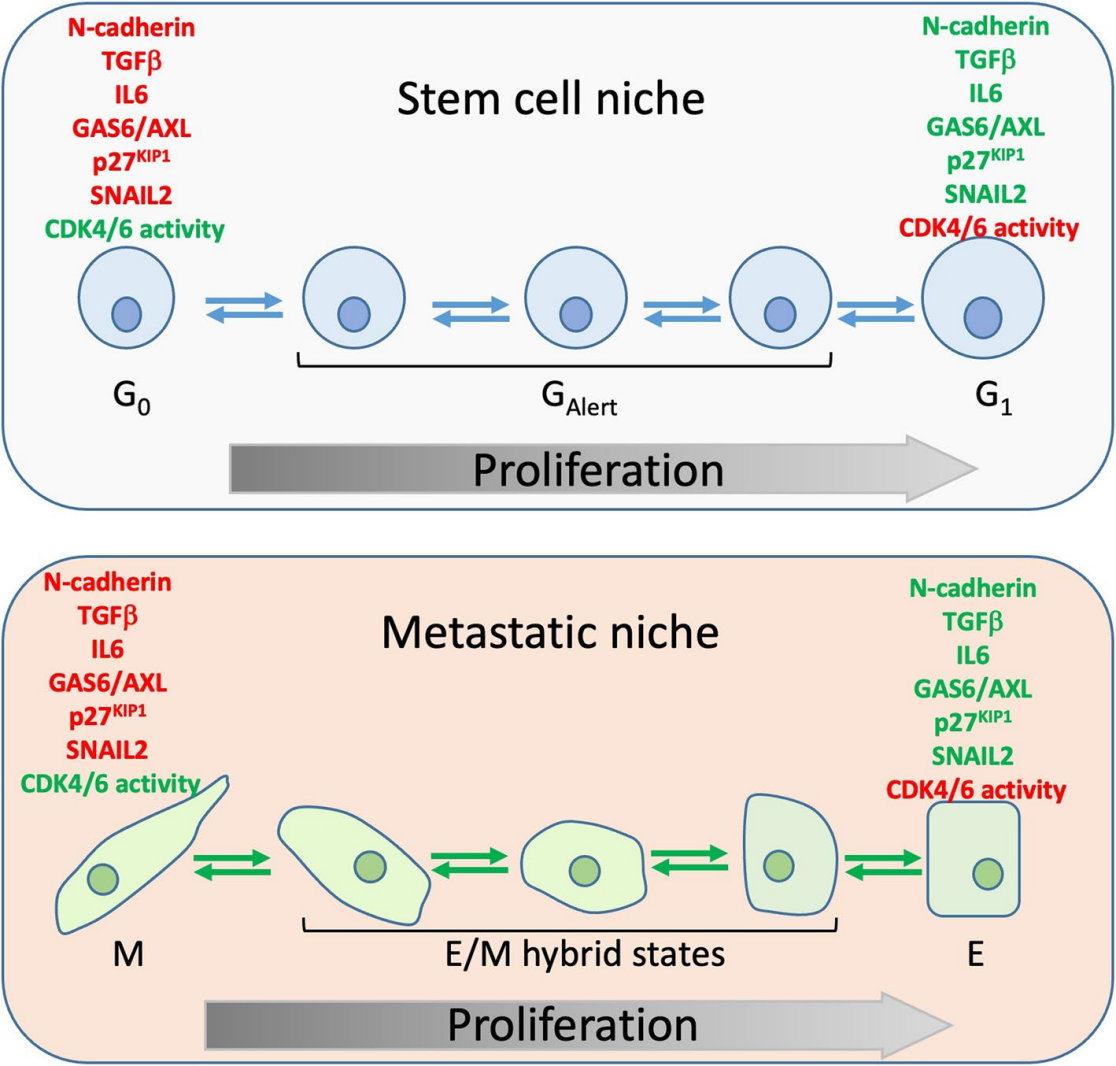
A parallel between G_Alert_ and E/M hybrid states of cells. The same factors such as N-cadherin, TGFβ, IL6, GAS6/AXL, p27^KIP1^ and SNAIL2 are implicated in both stem cell quiescence and EMT/MET equilibrium

To maintain quiescence/proliferation balance, stem cells communicate with each other and other components of a niche via cell–cell, cell-soluble factors, and cell-extracellular matrix (ECM) interactions [100, 101]. In healthy tissues, lack of niche-derived soluble factors is a determinant of quiescence, whereas secretion and release of growth factors from ECM enforce stem cells to enter the cell cycle. For instance, the presence of the hepatocyte growth factor (HGF) in injured muscles stimulates muscle cell proliferation via the c-MET/mTOR pathway and tissue repair [104]. Intercellular interactions are yet another factor supporting the quiescent state of the ASC. Quiescent ASC present in neural, muscle, hematopoietic, and endometrial stem cell niches are associated with the surrounding cells via AJ mediated by N-cadherin [105–108] and N-cadherin depletion leads to the dissolution of AJ, the release of stem cells from contact inhibition and their activation. In addition, niches maintain stem cell quiescence by producing growth-inhibitory factors, at least two of which, TGFβ1 and IL-6 family, are classical inducers of EMT programs [109, 110]. Therefore, at least two considerations, a role for the mesenchymal marker N-cadherin and the presence of the EMT triggers in the stem cell niches, indicate that in normal physiological conditions, EMT and stemness are associated (see Fig. 3).

The interconnection between EMT and cell stemness is apparent in embryonic tissues: NCCs, generated via EMT, represent a stem cell population that gives rise to various tissues in a developing embryo. In adult tissues, the impact of EMT programmes on stem cell biology has been studied in several types of epithelia. In the stratified epithelium of a mammary gland, stem cells are present within the outer basal layer. These stem cells express low levels of asymmetrically distributed E-cadherin that mediates their physical association with luminal cells. At the same time, adult mammary stem cells express some levels of mesenchymal markers N-cadherin and vimentin indicating that they reside in an intermediate phenotypic state in which epithelial and mesenchymal features are combined [111, 112]. This state is governed by the EMT-TF SLUG in conjunction with its interacting partner chromatin modifier Lysine-specific histone demethylase 1 (LSD1) [113]. Indeed, *SLUG* ablation in KO mice significantly delays mammary gland morphogenesis reflecting impaired lineage commitment, and the combined ectopic expression of SLUG and SOX9 results in the reprogramming of luminal cells into mammary stem cells with the renewal potential [114]. The role of a particular EMT-TF, SLUG, in stemness is not restricted to the mammary gland. In the stratified epithelium of other tissues, such as the prostate gland, upper airways of the lungs, and hair follicles, SLUG was shown to inhibit differentiation and support oligopotency [112, 115–117]. In addition to SLUG, other EMT-TFs, such as ZEB1, are expressed and sustain stemness in the subpopulations of basal cells in the prostate and mammary glands [118, 119].

As EMT-TFs are implicated in the biology of ASC, the Go-or-Grow principle might be applicable to these cell types. A recent study has shown that ZEB1 is required for branching morphogenesis and maintains quiescence in the subset of basal mammary stem cells via the upregulation of WNT pathway inhibitor Axin2 [120]. Likewise, experimental data support the existence of a Go-or-Grow dichotomy in HSC. The SDF-1 (stromal-derived factor-1) plays a crucial role in mobilisation and homing of hematopoietic stem cells via the interaction with cognate receptor CXCR4 [121]. Conditional ablation of either the receptor or the ligand stimulates HSC to enter the cell cycle [122], suggesting that this pathway is responsible for cell quiescence and motility. Several small Rho GTPases regulate cytoskeletal dynamics and cell motility downstream of SDF-1/CXCR4 signalling, while CDC42 is responsible for the retention of HSC in G_1_/G_0_ [123, 124]. Interestingly, CXCR4 is the effector of ZEB2 in some hematopoietic cells [125], and genetic ablation of *ZEB2* in hematopoietic lineage resulted in an increase in HSC and progenitors in the bone marrow and other specific features resembling myeloproliferative diseases in humans [126].

As opposed to the broad implication of SLUG in cell stemness in stratified epithelia, neither this protein nor other EMT-TFs are associated with stem cells in most single-layered epithelial tissues, such as pancreatic ducts or intestinal epithelium [112]. This is, however, in stark contrast with the cancer stem cells (CSC), cells that harbour stem features and are present in cancers derived from the same types of simple epithelia.

### EMT and cancer stem cells

CSC represent a distinct pool of tumour cells, which are highly tumourigenic but retain some features of normal epithelial stem cells. Like their normal counterparts, CSC are characterised by asymmetric division, self-renewal, and the ability to generate more differentiated progeny composing tumour mass. Because of their high tumourigenic potential, and resistance to various therapies, it is accepted that CSC represent a source for distant metastases and post-therapy cancer relapse. A causative link between EMT and cancer stemness was proposed theoretically and then demonstrated experimentally in a series of seminal works [127–131]. Expression of EMT-TFs SNAIL or TWIST1 in immortalised mammary epithelial cells generated cells with the CD44^high^CD24^−^ phenotype. These cells were capable of forming mammospheres, which contained both basal and luminal precursors. Similar mammosphere-forming CD44^high^ and CD24-negative cells with EMT gene expression signatures were isolated from normal and cancerous breast tissues [130].

The use of single-cell RNA sequencing technology for the analysis of the metastatic process in a PDX breast cancer mouse model has shown that EMT-derived stem-like cells appear at the early stages of metastatic dissemination [132]. These metastasis-inducing cells with reduced levels of E-cadherin and CD24 expressed EMT-TFs SLUG and TWIST1 and dormancy-associated gene signature including high *CDKN1B* (p27^KIP1^). Metastatic cells in PDX models with a high metastatic burden demonstrated higher expression of luminal differentiation genes, *c-MYC*, and proliferative signatures [132]. These data are in line with the hierarchical model of cancer metastasis. The model suggests that dormant CSC disseminate at the initial stages of the metastatic process, while extensive proliferation and differentiation occur at the advanced stages. The hierarchical model also applies to colorectal cancer, where Lgr5 (Leucine-rich repeat-containing G protein-coupled receptor 5) receptor is a marker of stem cells in the normal intestinal epithelium [133]. Lgr5^+^ cells isolated from normal tissues, primary tumours, or metastases do not show any EMT features but are characterised by stem cell gene signatures. After depletion of Lgr5^+^ cells from primary Apc^min/+^;Kras^G12D^-induced tumours, Lgr5^−^ cells displayed activated c-Myc pathway and overrepresentation of genes implicated in cell-cycle progression [134]. The hierarchical model is supported by the analyses of the pools of CTC (circulating tumour cells) isolated from blood samples of breast, colon, or lung cancer patients. These cells are derived from epithelial tumours, possess tumour-initiating potential, and express mesenchymal markers [135–137].

As cited above, compelling evidence collected in different cancer models indicates that epithelial traits are required for cancer cells to re-establish tumour growth in target organs and accomplish the metastatic process [132, 134]. Indeed, in the polyomavirus middle T antigen breast cancer mouse model, single metastatic tumor cells arriving at target organs were predominantly mesenchymal, but all micrometastases comprising three or more cells were E-cadherin-positive [138]. Irreversible loss of epithelial differentiation by enforced SNAIL expression led to the inhibition of tumorigenic and metastatic potentials in prostate and bladder cancer cells [139]. The role of E-cadherin was studied in mouse models of invasive ductal mammary adenocarcinoma. Although down-regulation of the *CDH1* gene coding for E-cadherin enhanced the invasive capacity of tumour cells, their metastatic potential was diminished as the result of activated TGFβ signalling, decreased cell proliferation, and reduced stress resistance [140]. These and many other reports demonstrate the importance of the reversibility of EMT programmes (EMT/MET plasticity) in metastatic processes [4, 112, 141]. While the epithelial state mainly contributes to the increasing mass of primary and secondary tumours (“Grow” function), the mesenchymal state facilitates invasion and colonisation (“Go” function). Indeed, given the tiny diameter of microcapillary utilized by metastasizing cells, loss of epithelial adhesion would make intravasation much more efficient. However, research combining in vivo imaging and lineage tracking has shown that migration of epithelial cell clusters is often a common feature. In these clusters, cells retain intercellular junctions and form cohesive migrating groups (so-called collective migration) [142]. Regardless of their epithelial appearance, intercellular adhesion in these groups is weakened because of a partial EMT [143] (see next section). Some reports have shown that MET programs are essential not only for forming macrometastases but also capable of generating highly tumourigenic epithelial-like CSC with metastasis-seeding properties [144–146]. Moreover, in some cases, such as the syngeneic mouse model of breast cancer, epithelial CTCs (defined by high levels of Epcam, E-Cadherin, and Grhl2 expression) have the strongest metastatic ability as compared to the pool of mesenchymal CTCs (low EpCAM, E-cadherin expression, high levels of Vimentin, and EMT-TFs expression) [147].

### Intermediate EMT stages and reconciliation of “Go” and “Grow” features

A concept reconciling findings that “Go” and “Grow” capabilities are combined in a single cell proposes that EMT and MET do not represent binary switches. This model suggests that both programs represent processes empowering cancer cells by the spectra of both epithelial and mesenchymal traits leading to the generation of so-called hybrid, quasi-mesenchymal, or E/M phenotypes. In vitro studies have shown that several distinct intermediate states can be delineated, which differ with regard to the expression of differentiation markers [148–150]. These distinct E/M states are characterised by particular chromatin landscapes and are epigenetically stabilized to a certain degree. However, cells in the early and late hybrid states were the most plastic and retained the capability to equally produce epithelial and mesenchymal subpopulations [151–153].

The in vivo existence of E/M cells was demonstrated by immunohistochemistry or by the analyses of gene expression signatures. Significantly, enrichment for hybrid EMT RNA signature has been associated with poor survival and resistance to therapy in several tumor types (reviewed in: [151]), including breast cancer [145]. Whereas quiescent CD44^high^/CD24^−^ cells with EMT signatures were detected primarily at the invasive fronts of primary mammary tumours, proliferative epithelial cells expressed aldehyde dehydrogenase (ALDH^+^) and were located within central tumour areas. A pool of the cells with intermediate E/M features, CD44^high^/CD24^−^/ALDH^+^ cells, was also detected in tumour tissue and corresponded to less than 0.1% of cells within a tumour [145]. In later studies, the isolation of E/M cells from mammary carcinoma cell cultures was carried out using a combination of CD104 and CD44 antigens. These cells stably resided in the hybrid state, displayed CSC features, and expressed high levels of SNAIL, SLUG, and TWIST1. The canonical WNT signalling pathway drove this highly aggressive phenotype. Ectopic expression of ZEB1 in E/M cells induced a complete EMT, inhibited tumourigenicity, and triggered a switch from canonical to non-canonical WNT signalling [154]. These results show that although individual EMT-TFs are capable of inducing complete EMT in vitro, their functions in tumourigenesis are different in vivo. Indeed, ZEB1, but not TWIST1 or SNAIL, is a driver of tumour progression in the mouse model of pancreatic ductal adenocarcinoma Kras^G12D^;P53R^172H/+^ [155, 156]. A different series of cell surface markers (EpCAM, VCAM, ITGAV, and ITGB3) along with single-cell RNA sequencing (sc-RNAseq) was applied to identify E/M states in squamous cell carcinoma of the skin [152].

Application of unbiased single-cell RNA sequencing approach led to the identification of intermediate states in different cancer types, including glioblastoma, metastatic breast cancer, pancreatic ductal adenocarcinoma, squamous cell carcinoma of the skin, head and neck, and cutaneous melanoma [151, 157–160]. In particular, an intermediate state in malignant melanoma exhibited an NCC transcriptional program, which overlapped with the expression programs specifying proliferative, invasive and CSC phenotypes. These cells driven by the nuclear receptor RXRG were present in the pool of persister cells tolerant towards BRAFV600E and MEK inhibitors [158].

E/M cells were present in CTC pools isolated from the blood samples of patients with various cancer types, and E/M CTC, rather than fully differentiated epithelial or mesenchymal cells, are associated with short metastasis-free and overall survival [151]. Epithelial cells expressing mesenchymal markers are often detected in clusters of CTC. These clusters form microemboli via association with non-malignant cells, including the platelets, which maintain mesenchymal features of CTC by releasing TGFβ [161]. Circulating microemboli have up to 50 times more metastatic potential than single CTCs [162]. These clusters are oligoclonal in their origin and do not contain intravascular CTC aggregates [163]. In breast and pancreatic cancer modes, the CTC clusters are composed of highly motile E/M and proliferative epithelial cells. They are capable of Plakoglobin- and E-cadherin-dependent collective migration and enter the circulation as groups while retaining proliferative potential [143, 164].

Thus, analyses of animal models and patient samples show that combining “Go” and “Grow” capabilities in E/M hybrid states facilitates tumorigenesis. Stimulating one capability at the expense of the other locks cells in E or M states (for instance, via manipulation with the expression levels of ZEB1) [154, 155] and reduces tumorigenicity and metastasis.

### E/M plasticity, exit from quiescence and metastasis

While most CTCs do not survive in circulation, a proportion extravasate into target organs, form pools of disseminated tumour cells (DTC), and survive therapy in a reversible dormant state. The presence of dormant cancer cells detectable in bone marrow reflects a condition termed the minimal residual disease that is a sensitive indicator of poor prognosis and a major clinical challenge. Understanding the mechanisms of the exit from dormancy is of tremendous importance also because the colonisation of secondary organs is the bottleneck in the metastatic process [165]. Three likely complementary mechanisms are implicated in maintaining single-cell or micrometastatic dormancy: angiogenic dormancy, immune-mediated dormancy, and cellular dormancy [166, 167]. Whereas the two first scenarios involve a balance between cell proliferation and death imposed by lack of nutrients or immune attacks, the third scenario suggests that DTC may enter deep quiescence. Cell cycle exit is associated with resistance to anti-proliferative drugs; quiescent DTCs evade immunosurveillance [168] and are insensitive to immunotherapies. Therefore this third type of dormancy can be particularly clinically relevant and will be discussed below.

By combining a sophisticated lineage-tracing approach with the scRNA-seq, Simeonov and colleagues investigated mechanisms of metastasis in PDAC and identified four E/M hybrid stages (H1-H4) in addition to the fully differentiated epithelial and mesenchymal cell pools [160]. In this Kras^G12D^;P53R172^H/+^ -driven cancer model, proliferative gene expression signatures were observed in cells with the E and H3 differentiation states. Based on the expression of cell surface markers, EpCAM-negative squamous carcinoma cells were separated into six discrete subpopulations of the E/M cells presenting different degrees of mesenchymal differentiation [151, 152]. In agreement with the Go-or-Grow conception, the proliferative capability gradually decreased while the invasive potential progressively increased along the epithelial-mesenchymal spectrum. In the experimental metastasis assay, early E/M cells (i.e., E/M cells positioned closer to the epithelial end of the spectrum) exhibited the highest metastatic potential that significantly exceeded the metastatic propensity of fully differentiated E or M cells [151]. Likewise, in the Cre^+/-^*Pten*^L/L^;*Kras*^G12D/+^ mouse model of prostate cancer, E/M hybrid cells exhibited increased proliferative and decreased invasive potential compared to highly invasive mesenchymal cells [169].

Cells corresponding to the mesenchymal end of the E/M spectrum are highly invasive and, due to their non-proliferative nature, apoptosis-resistant. Therefore, it has been accepted that these cells are the source of dormant DTCs, and supposedly they represent metastatic seeds. The ability to differentiate along the mesenchymal-epithelial axis leading to the exit from dormancy is the requirement for successful colonisation. This ability depends on the mutational landscape of tumour cells and is driven by microenvironmental cues.

DTCs reside in metastatic niches, specific locations in target organs, which provide anchorage and survival support [170]. There is an apparent analogy between metastatic niches occupied by DTCs possessing the CSC features and ASC niches, which are present in most normal tissues. The pre-existing ASC niches can attract metastatic tumour cells. For example, HSC niches in the bone marrow are targeted and occupied by DTCs derived from metastatic prostate cancer [171, 172]. Apparently, the dormant state of HSC and DTCs is controlled via the same pathways initiated by niche-produced ligands belonging to the TGFβ family [172]. Additionally, growth arrest specific-6 (GAS6), a ligand secreted by osteoblasts in the bone marrow environment, contributes to the dormancy of prostate cancer-derived DTCs and inhibits the proliferation of hematopoietic progenitors [172, 173]. GAS6 and the cognate receptor AXL, a TAM (TYRO3, AXL, MER) RTK family member, play a major role in EMT, therapy resistance, and metastasis in different tumour types [174]. Shifting the balance in HSC niches towards quiescence and more mesenchymal end of the E/M spectrum aligns with multiple reports describing the role of GAS6/AXL signalling in EMT and drug resistance in cancer [174, 175]. Interestingly, the analysis of neural stem cells showed that AXL and MER were part of the 1000 gene signature that is downregulated in cells, which were released from quiescence and entered the cell cycle [176]. These observations indicate that TAM receptors maintain quiescence in different lineages, which is in line with the widespread role of these molecules in drug resistance in cancer. In breast and ovarian cancer, AXL-induced EMT programmes are mediated by SNAIL family members [63, 177]. Remarkably, SLUG was shown to maintain quiescence also in HSC in bone marrow niches [178]. However, it remains unclear if, by analogy with EMT-inducing malignant signalling, SLUG is an effector of the GAS6/AXL pathway in normal stem cells as well.

The newly discovered facets of ASC reveal new aspects of their similarity with DTCs. Quiescence of ASC does not represent a deep static condition of dormancy but is highly dynamic. Quiescent ASC may transit between different states, characterised by various depths of dormancy [100]. For example, in addition to the canonical deep quiescence (G_0_), muscle ASC can acquire another form of quiescence, G_Alert_. Cells in the G_Alert_ phase require much less time than canonical G_0_ cells to enter the cell cycle in injured muscle tissue. G_0_/G_Alert_ transition is regulated by the mTORC1 pathway during the repair of injured tissue [179, 180]. Likewise, the phenomenon of fluctuating depths of quiescence was reported in hematopoietic ASC [181]. In contrast to G_0_/G_Alert _transitions, the balance between different phases of quiescence in hematopoietic ASC was independent of mTORC1 and regulated by the levels of CDK6. Apparently, Rb/E2F pathway regulates transitions within G_0_, as well as the transition from the quiescence to G_1_ [100, 182]. As the G_0_ phase is characterised by much higher levels of p27^KIP1^ than G_1_ [183], this CDK inhibitor is likely implicated in G_0_/G_1_ transition by regulating CDK4/6 activity. However, the detailed mechanism of this transition is not studied, definite G_0_ markers are unknown, and this area warrants further investigations.

The plastic nature of quiescent ASC is reminiscent of tumour cells which have the potential to adopt various differentiation states along the E/M spectrum. This plastic feature of both cell types is predetermined by characteristic epigenetic patterns. Trimethylated lysines 4 and 27 of histone 3 (H3K4me3 and H3K27me3) mark promoters of actively transcribed and silenced genes, respectively. In some populations of ASC identified in muscle and neuronal tissues, both repressive and activating marks are present in the same chromatin domains (so-called bivalent domains) [184, 185]. This organisation of the chromatin allows rapid activation or silencing of genes by removal of H3K27me3 or H3K4me3 marks, respectively, in response to the proliferation or differentiation-inducing stimuli. Remarkably, the same pattern of chromatin modifications was associated with E/M plasticity [186, 187]. The similarity in the epigenetic landscapes between ASC and disseminated tumour cells is an indication that common signalling mechanisms govern the exit from quiescence in stem cell and metastatic niches. This assumption can be tested by analyzing similarities in the chromatin accessibility landscapes in ASC and cancer cells derived from the same tissue and undergoing G_0_-G_Alert_-G_1_ and E-E/M-M transitions, respectively.

## Concluding remarks and perspectives

Uncontrolled proliferation, enhanced invasiveness, and drug resistance of dormant cancer cells are critically important from a clinical perspective. In this review, we discussed the migration—proliferation dichotomy (“Go-or-Grow” conception) in light of EMT/MET plasticity. EMT and MET programs have been implicated in balancing invasive and proliferating states and also in the acquisition of stem cell traits in both homeostasis and cancer. In recent years, the identification of cells combining epithelial and mesenchymal features led to the re-evaluation of a concept considering EMT and MET as binary switches between fully differentiated states. Cells adopting intermediate states express both epithelial and mesenchymal markers. They were isolated from stem cell compartments of adult epithelial tissues, primary tumours, and populations of CTCs. Thus, EMT/MET plasticity is a characteristic feature of both DTC and ASC.

ASC often reside in the dynamic G_Alert_ phase, which is important for accelerated regeneration of damaged tissues. It remains not studied whether DTC undergo a similar type of G_0_-G_Alert_-G_1_ transitions in the process of colonisation and during cancer relapse after therapy failure. Given the established role of EMT triggers in regulating ASC proliferation, it is reasonable to speculate that the molecular pathways controlling the transition across the E-M axis are also implicated in the dynamic shifts within the G_0_ phase. Then the interpretation of the signals in the niches will involve coordinated changes in signalling networks controlling cell motility and cell cycle entry leading to the “Go-or-Grow” decision.

Cell plasticity is responsible for cellular adaptations to microenvironmental cues during the metastatic process and for generating drug-tolerant persister cells. The presence of bivalent domains in the chromatin of cancer cells is critical for determining their multipotency. Therefore, targeting chromatin bivalency can limit the cellular ability to transit across the E/M axis and to adopt characteristics of G_Alert_ states. There is a great hope that further research in cancer epigenetics and the development of tools for targeting bivalent chromatin will freeze cancer cells in the drug-susceptible state and prevent metastasis.

## Data Availability

Not applicable.

## Abbreviations

EMT: Epithelial mesenchymal transition
MET: Mesenchymal epithelial transition
AJ: Adherens junctions
EMT-TFs: Epithelial mesenchymal transition transcription factors
MET-TFs: Mesenchymal epithelial transition transcription factors
OVOL2: Ovo-Like Transcriptional Repressor 2
GRHL2: Grainyhead-like protein
LDL2: Lethal Giant Larvae 2
KLF4: Krüppel-like factor 4
CLDN4: Claudin-4
CLDN5: Claudin-5
lncRNAs: Long non-coding RNAs
ceRNAs: Competing endogenous RNAs
CKIs: Cyclin-dependent kinase inhibitors
AC: Anchor cells
NCC: Neural crest cells
ASC: Adult stem cells
HSC: Hematopoietic stem cells
ECM: Cell-extracellular matrix
HGF: Hepatocyte growth factor
LSD1: Lysine-specific histone demethylase 1
SDF-1: Stromal-derived factor-1
CSC: Cancer stem cells
Lgr5: Leucine-rich repeat-containing G protein-coupled receptor 5
CTC: Circulating tumour cells
ALDH +: Aldehyde dehydrogenase
sc-RNAseq: Single-cell RNA sequencing
DTC: Disseminated tumour cells
GAS6: Growth arrest specific-6
